# *C. elegans* expressing D76N β_2_-microglobulin: a model for *in vivo* screening of drug candidates targeting amyloidosis

**DOI:** 10.1038/s41598-019-56498-5

**Published:** 2019-12-27

**Authors:** Giulia Faravelli, Sara Raimondi, Loredana Marchese, Frederick A. Partridge, Cristina Soria, P. Patrizia Mangione, Diana Canetti, Michele Perni, Francesco A. Aprile, Irene Zorzoli, Elia Di Schiavi, David A. Lomas, Vittorio Bellotti, David B. Sattelle, Sofia Giorgetti

**Affiliations:** 1 Department of Molecular Medicine, Institute of Biochemistry, University of Pavia, 27100 Pavia, Italy; 2 Centre for Respiratory Biology, UCL Respiratory, Division of Medicine, University College London, Gower Street, London, WC1E 6JF United Kingdom; 3 Wolfson Drug Discovery Unit, Centre for Amyloidosis and Acute Phase Proteins, University College London, London, UK; 4 Centre for Misfolding Diseases, Department of Chemistry, University of Cambridge, Cambridge, CB2 1EW UK; 5 Institute of Biosciences and Bioresources (IBBR), CNR, 80131 Naples, Italy

**Keywords:** Biochemistry, Biological techniques

## Abstract

The availability of a genetic model organism with which to study key molecular events underlying amyloidogenesis is crucial for elucidating the mechanism of the disease and the exploration of new therapeutic avenues. The natural human variant of β_2_-microglobulin (D76N β_2_-m) is associated with a fatal familial form of systemic amyloidosis. Hitherto, no animal model has been available for studying *in vivo* the pathogenicity of this protein. We have established a transgenic *C. elegans* line, expressing the human D76N β_2_-m variant. Using the INVertebrate Automated Phenotyping Platform (INVAPP) and the algorithm Paragon, we were able to detect growth and motility impairment in D76N β_2_-m expressing worms. We also demonstrated the specificity of the β_2_-m variant in determining the pathological phenotype by rescuing the wild type phenotype when β_2_-m expression was inhibited by RNA interference (RNAi). Using this model, we have confirmed the efficacy of doxycycline, an inhibitor of the aggregation of amyloidogenic proteins, in rescuing the phenotype. In future, this *C. elegans* model, in conjunction with the INVAPP/Paragon system, offers the prospect of high-throughput chemical screening in the search for new drug candidates.

## Introduction

β_2_-microglobulin is a protein composed of 99 amino acids with a characteristic β-sandwich immunoglobulin fold. It serves as the light chain of the class I major histocompatibility complex. Amyloid formation by wild-type β_2_-m occurs as a severe complication of long-term dialysis for patients with end-stage kidney failure, as a result of the persistently high plasma concentration of β_2_-m^1^. So far, only one genetic form of β_2_-m related amyloidosis has been described and this is caused by the mutation Asp76Asn. The clinical and experimental work carried out on this variant has advanced our current understanding of amyloidogenesis of β_2_-m and other globular proteins, including the role of biomechanical forces in protein misfolding^2^ as well as the mechanism of β_2-_m fibrillogenesis^1,3^.

Whereas the mechanism of misfolding and aggregation *in vitro* has been extensively clarified, the mechanism of toxicity of amyloid in general and of β_2_-m in particular remains an unmet challenge. Animal models offer important insights on the identification of the protein species causing the tissue toxicity. However, attempts to generate animal models for β_2_-m associated diseases have stalled as, in spite of good levels of expression of β_2_-m in transgenic mice expressing the wild type^4^ protein or the pathogenic variant D76N, no pathological phenotype of β_2_-m amyloidosis has been observed.

The nematode genetic model organism *Caenorhabditis elegans* (*C. elegans*) offers the prospect of studying the effects of the β_2_-m variant *in vivo*. *C. elegans* models have already been successfully deployed to characterize important molecular aspects of other protein aggregation diseases such as Alzheimer’s, Parkinson’s and Huntington’s diseases^5–9^. *C. elegans* has several advantages. It shares many characteristics with mammalian systems with respect to genetics, biochemical and physiological functions. It is small, transparent and a self-fertilizing hermaphrodite producing 300 identical progeny in 3 days; its life cycle is short (2–3 weeks) and it is easy and inexpensive to maintain in the laboratory^10^. Moreover, chemical and genetic screenings in *C. elegans* are becoming an established method to search for new therapeutic tools and to uncover and validate drug targets^11^. We have previously described the phenotypes of transgenic worm strains transiently expressing wild type β_2_-microglobulin and some isoforms not related to the familial form of the disease^12^. As evidence of the reliability of the *C. elegans* model, we observed that the severity of proteotoxicity *in vivo* correlated well with the protein’s amyloidogenic propensity measured *in vitro*.

In this paper, we characterize a new transgenic *C. elegans* strain, named CPV27, expressing the D76N variant, in which the β_2_-m gene is integrated into the *C. elegans* genome. The development of this new strain was challenging, due to the high toxicity of the expressed protein at the embryonic stage of development. We have therefore prepared a strain in which the expression of the disease-causing variant is under temperature-dependent control, using the well-characterized mRNA-surveillance system of *C. elegans*^13^. This new worm strain is the first robust animal model for studying *in vivo* the effects of the pathogenic variant of D76N β_2_-m and represents a valuable new tool with which to explore further the disease mechanism and search for new drug candidates to combat β_2_-m amyloidogenesis.

## Results

### Expression of the D76N β_2_-m variant

**Figure 1:**
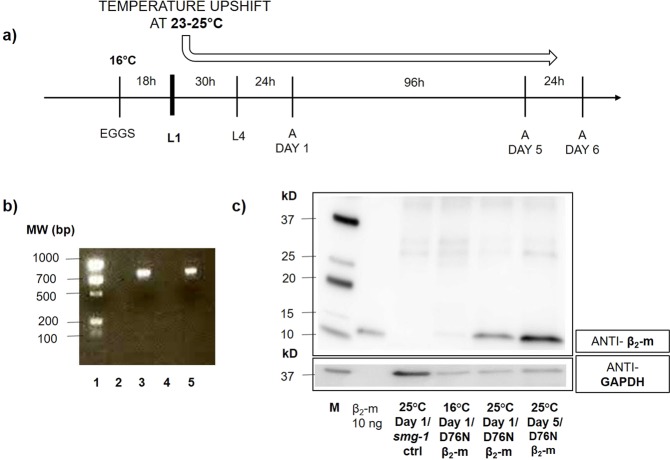
D76N β_2_-m *C. elegans* strain genotype characterization and β_2_-microglobulin expression. **(a)** Scheme of *C. elegans* manipulation highlighting the stage in which the β_2_-m expression is activated by temperature switch. Eggs were maintained at 16 °C and L1 larvae were up-shifted to 23–25 °C in order to induce the expression of the protein. **(b)** PCR genotyping of adult transgenic nematodes. The expected size of PCR products (about 800 bp) was observed after DNA electrophoresis on 1.5% agarose gel (lane 1: EZ Load Precision, BioRad, lane 2,4: pcr products of DNA extracted from *smg-1* (cc546) ancestral strain, lane 3,5: pcr products of DNA extracted from D76N β_2_-m worms. **(c)** Representative western blot of β_2_-m expression. Equal amounts of protein (10 μg) were loaded for each sample from *smg-1* (-ctrl) or D76N β_2_-m expressing nematodes and immunoblotted with polyclonal anti-human β_2_-m antibody (Dako) and anti-GAPDH antibody used as loading control (M = Molecular weight standard: Precision Plus Western C, BioRad). Uncropped scans of immunoblots are shown in Supplementary Fig. S1.

We previously reported a *C. elegans* model of wild type β_2_-m amyloidosis in which the protein was expressed in the body-wall muscles under the control of the promoter of the myosin gene *unc-54*^12^. However, our attempts to express the D76N β_2_-m variant in *C. elegans* under the same promoter failed because the nematodes died before reaching the first larval stage, likely due to the high toxicity of the protein variant (data not shown). To bypass the lethal effect of such constitutive protein expression, we adopted the well-characterized *smg* inducible system^13^. In this protocol, at the permissive temperature of 16 °C, the mRNA of the transgene is readily degraded, whereas an upshift to a non-permissive temperature that is still compatible with the normal growth of the worms (23–25 °C) (Fig. 1a), inactivates the surveillance machinery allowing stabilization and translation of the transgene mRNA. The *smg* system enabled us to switch-on the expression of D76N β_2_-m at the first larval stage (L1), thereby avoiding protein synthesis and its related toxic effects in the embryonic stage.

Transgenic *C. elegans* were engineered to express D76N β_2_-m in bodywall muscles under the promoter of another myosin gene, *myo-3*. As the amyloid is an extracellular protein deposit, we sought to obtain protein secretion by replacing the previously used human β_2_-m signal peptide^12^ with the signal peptide of the endogenous *sel-1* protein^14^. We have used, as a co-injection marker, the p*odr-1::rfp* plasmid, which shows a strong pattern of red fluorescent protein (RFP) expression (RFP expressed in two head neurons, AWB and AWC). After engineering the D76N β_2_-m expressing plasmid, microinjection into the gonad and integration of the transgene by UV irradiation, the exact genotype of D76N β_2_-m expressing nematodes was confirmed by single worm PCR (Fig. 1b, lane 3,5) and DNA sequence analysis.

The strain obtained after integration *smg-1(cc546); pavIs1[pmyo-3::SPsel-1::hD76Nβ*_*2-*_*m::Smg sensitive 3*′*UTR; podr-1::rfp]*, named CPV27, correctly expressed D76N β_2_-m, as shown by western blot analysis (Fig. 1), when animals were grown at 25 °C beginning at the L1 larval stage. Levels of protein increased from the first to the fifth day when nematodes were grown at 25 °C (Fig. 1c). D76N β_2_-m nematodes, maintained at 16 °C and collected 24 h after the L4 stage, were also analyzed. The absence of expression of β_2_-m at 16 °C confirmed the efficiency and the specificity of the thermo-inducible system, therefore the *smg-1(cc546)* ancestral strain (PD8120 strain) served as a negative control (Fig. 1c).

### Aggregation state of the β_2_-m variant

**Figure 2:**
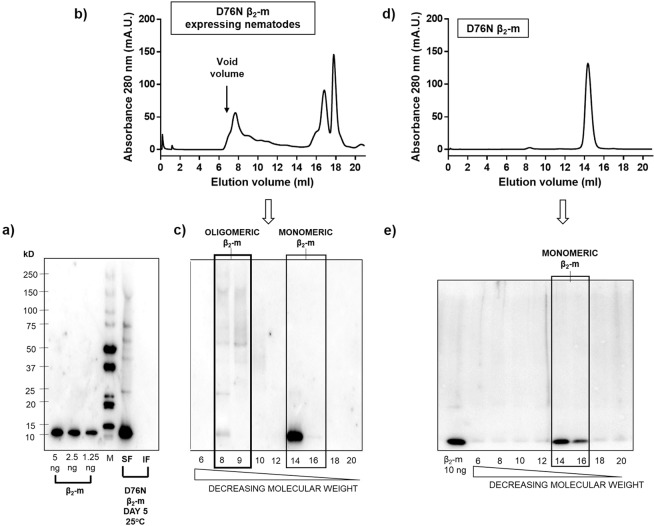
Self-assembly of β_2_-m D76N in transgenic *C. elegans* strain. **(a)** Immunoblot analysis of soluble (SF) and insoluble fraction (IF) of worm lysates grown at 25 °C resolved via 8–18% SDS PAGE and detected with anti-β_2_-m antibody (DAKO). The insoluble fraction was washed twice in PBS buffer before loading onto gel. (M = Molecular weight standard: Precision Plus Western C, BioRad). **(b–d)** Size-excluded soluble proteins from β_2_-m expressing nematodes and recombinant D76N β_2_-m. Shown is the absorbance at 280 nm (Abs 280) of eluted material against elution volume. Fractions (1 ml) were collected. **(c–e)** Immunoblot analysis of size-excluded fractions (6–20) of β_2_-m expressing worms’ lysates and recombinant β_2_-m resolved via 8–18% SDS PAGE and detected with anti-β_2_-m antibody (DAKO). Uncropped scans of immunoblots are shown in Supplementary Fig. S2a.

A search of amyloid deposits was carried out by microscopic analysis of worms stained with the amyloid probe NIAD-4 as previously described^15^. However, we did not detect amyloid material at any stage of development (data not shown). In spite of a lack of clearly visible amyloid deposits, the β_2_-m variant in worms displayed a high propensity to form soluble self-assemblies as shown by both electrophoresis and size exclusion chromatography (Fig. 2). Worms were lysed and after centrifugation, both supernatant and pellet were first analysed by SDS-PAGE and β_2_-m detected by immunoblotting. As clearly shown in Fig. 2a, almost all the β_2_-m present is recovered in the supernatant and not in the pellet obtained from the worm lysate.

Chromatographic analysis carried out on the supernatant in physiological buffer reveals that β_2_-m is eluted in a wide range of molecular weights, from the most abundant 11 kDa of the monomeric state (fractions 14–16, Fig. 2b,c) to heterogeneous oligomeric states up to and beyond 100 kDa (fractions 8–9, Fig. 2b,c). Such heterogeneous states of aggregation are not present when recombinant β_2_-m was loaded at different concentrations (Fig. 2a) and this finding is confirmed when the soluble fraction of recombinant β_2_-m is analysed by size exclusion chromatography (Fig. 2d,e). The *smg-1* mutant worms were analysed as well and used as negative controls in order to exclude any possible cross-reaction of the antibody (S2b).

Our hypothesis, which has been confirmed by the experiments on the characterization of CPV27 transgenic strain reported below, was that the expression and accumulation of monomeric and oligomeric species of D76N β_2_-m variant correlate with a pathological phenotype in our *C. elegans* strain.

### Effects of β_2_-m expression on the *C. elegans* phenotype

#### INVAPP/Paragon analysis of worms’ growth and motility

**Figure 3:**
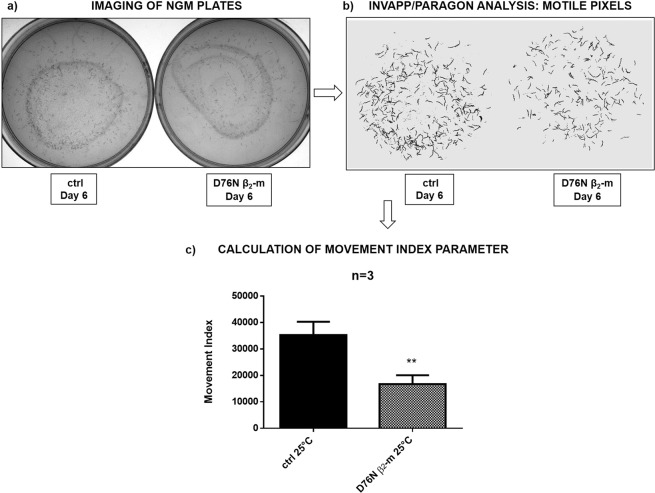
Movement index analysis using the INVAPP/Paragon system. 3 L4 worms/well were placed onto NGM 6-well plates, fed with OP50 *E. coli*, and maintained at 25 °C for six days **(a)**. Worms were imaged using INVAPP Paragon software **(b)**. Data are mean of movement index parameter ± SEM; which was obtained after processing the acquired images **(c)**. Three independent experiments were carried out and the results were plotted using GraphPad Prism (v6), **p < 0.01 vs the control (s*mg-1*) according to t-test.

To investigate the effect of expressing D76N β_2_-m on nematode growth and motility, an automated analysis was carried out using the recently developed INVAPP/Paragon system^16^. Briefly, the automated platform is able to quantify growth and/or motility of nematodes, by acquiring images of nematode growth medium (NGM) plates containing worms and by thresholding pixel variance to determine motion. Quantified movement is recorded as a movement index parameter, which increases with the number and size of worms per plate, reflecting a larger number of ‘motile’ pixels in the recording. After placing three L4 larvae per plate, we followed the growth and motility of their progeny. At day six at 25 °C (Fig. 3a), both the *smg-1* controls and D76N β_2_-m expressing worms were imaged with INVAPP/Paragon (Fig. 3b) and their movement index was measured (Fig. 3c). D76N β_2_-m expressing worms showed slower growth and reduced overall motility than the *smg-1* control strain. Indeed, the movement index was 53% of the value observed for the *smg-1* control strain (Fig. 3c, **p < 0.01, t-test). The INVAPP/Paragon platform shows that an abnormal behavioural phenotype is associated with the expression of the amyloidogenic protein.

**Figure 4:**
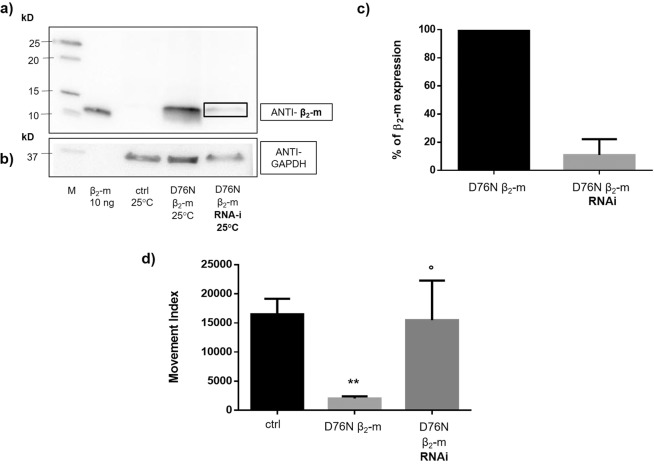
D76N β_2_-m expression is prevented by RNA-interference (RNAi). Equal amounts of proteins (40 µg) were loaded on each lane and immunoblotted with polyclonal anti-human β_2_-m antibody **(a)** and with anti-GAPDH **(b)**. Lanes: M = Precision Plus Western C (Bio Rad) molecular weight standard. s*mg-1* incubated at 25 °C and collected at day 1 of adulthood. D76N β_2_-m expressing nematodes incubated at 25 °C and collected at day 1 of adulthood and fed with HT115 bacteria transformed, as reported in the Methods section, with PAV2 plasmid for RNA-interfering (black box) or with control bacteria. Uncropped scans of immunoblots are shown in Supplementary Fig. S3. **(c)** Percentage of β_2_-m expression is given as D76N β_2_-m/GAPDH ratio of the WB band density of the RNAi strain relative to the control at their first day of adulthood. Density of the bands was determined by Image Studio Lite (LI-COR Biosciences). Three independent WB experiments were carried out and the results were plotted using GraphPad Prism (v6), p = 0.0052 vs the control level of expression (black) according to one sample t-test. **(d)** Index movement analysis. Three L4 worms were placed into NGM plates, fed with HT115/L4440 or HT115 bacteria transformed with PAV2 for RNA interference. Plates were maintained at 25 °C for 5 days. At this stage, the plates with adult worms and progenies were analysed by INVAPP Paragon software. Three independent experiments were carried out and the results were plotted using GraphPad Prism (v6). Data are mean of movement index parameter ± SEM; **p < 0.01 vs ctrl worms and °p < 0.05 vs the non-silenced D76N β_2_-m expressing according to one-way Anova.

To check whether the phenotype of the D76N β_2_-m strain correlated with the expression of the D76N variant of β_2_-microglobulin, we silenced the β_2_-m gene by feeding worms with bacteria that produce dsRNA targeting the β_2_-m transgene. Western blot analysis of worms treated with RNAi bacteria were compared with those fed with control bacteria (Fig. 4a,b) showing that the D76N β_2_-m protein was significantly reduced relative to the control (Fig. 4c). Most importantly, the reduction in the levels of β_2_-m expression correlated with the near complete abrogation of the D76N β_2_-m strain pathological phenotype (Fig. 4d, dark grey bar, n = 3, °p < 0.05 vs. not silenced D76N β_2_-m expressing worms, t-test).

The index movement parameter recorded by the INVAPP/Paragon system, combines the contributions of different phenotypic features: motility, larval development and size of the progeny.

In order to further dissect the results obtained with INVAPP/Paragon, we have carried out a body bends assay, a lifespan measurement and a brood size assay.

#### Body bends assay

**Figure 5:**
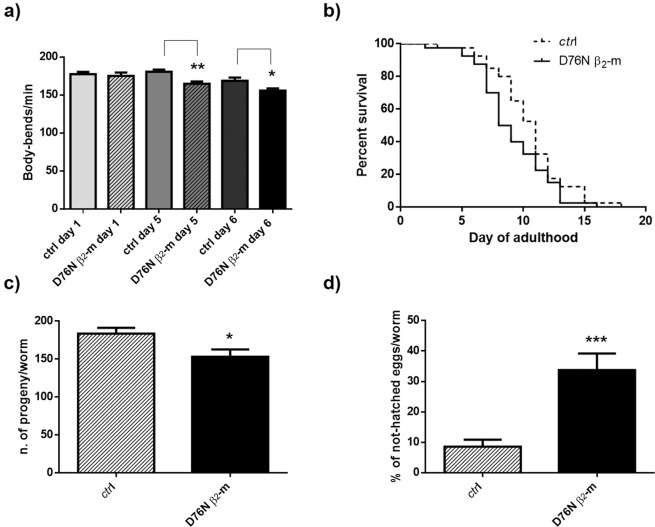
Characterization of the phenotype of D76N β_2_-m strain by classic behavioral assays. **(a)** Egg-synchronized control worms (*smg-1*) and D76N β_2_-m expressing worms were placed at 23 °C into fresh NMG plates seeded with OP50 *E*. *coli*. At day 1, 5 and 6 of adulthood body bends were scored in liquid. At least three independent assays were performed. Data are mean of number of body bends/min ± SEM; **p < 0.01 and *p < 0.05 vs the control strain (*smg-1*), according to one-way ANOVA (N = 40 animals for each group). **(b)** Kaplan–Meier survival curves of control nematodes and D76N β_2_-m strain. Data are expressed as mean of three independent experiments (N = 40 animals for each group, χ^2^ = 5.52, p = 0.019 according to Peto-Peto-Prentice test). **(c)** Total eggs deposition for s*mg-1* and D76N β_2_m strains maintained at 23 °C. At least three independent assays were performed. Error bars represent the SEM, *p < 0.05 vs. the control strain (*smg-1*) according to t-test. **(d)** Percentage of not-hatched eggs of *smg-1* and D76N β_2_-m strains analyzed 24 h after deposition. At least three independent assays were performed. Error bars represent the SEM, ***p < 0.001 vs. the control strain according to t-test.

The results of body bends assay at 23 °C are reported in Fig. 5a. At day 1 of adulthood, there is no difference between D76N and controls. A statistically significant reduction in the number of body bends per minute is observed from the fifth day (about 8% decrease; n = 3, **p < 0.01 at day 5 and *p < 0.05 at day 6 when compared to corresponding *smg-1* controls, one-way ANOVA, N = 40 animals for each group). The body bends assay shows that the phenotypic abnormality of D76N β_2_-m expressing worms, quantified using the INVAPP/Paragon system, was only partially due to dysfunctions in coordination and motility. For this reason, other phenotypic assays were performed.

#### Lifespan

Measurement of lifespan was performed on synchronized nematodes upshifted to 23 °C from the first larval stage. The D76N β_2_-m strain showed a median survival of 8 days, while that of the *smg-1* control strain was 10 days (Fig. 5b). Apparently, the difference between the two strains is mostly attributable to the first part of the Kaplan-Meier graph, and after ten days of adulthood, the two plots become closely aligned. A Peto-Peto-Prentice test shows that these differences are significant (χ^2^ = 5.52, p = 0.019).

#### Brood size assay

Brood size assay of transgenic animals was then performed by counting the number of eggs laid from a single nematode during the first 48 h of adult life at 23 °C (Fig. 5c, n = 3 *p < 0.05 vs *smg-1* control strain according to t-test). The D76N β_2_-m expressing animals showed a 17% reduction in brood size compared to the *smg-1* control strain (Fig. 5c). In addition, the egg viability test was performed, by counting the number of unhatched eggs 24 h after their deposition (Fig. 5d, ***p < 0.001 vs. the control strain, t-test). When compared to the control strain, egg viability was significantly reduced in the D76N β_2_-m expressing strain: *smg-1* control nematodes had less than 10% unhatched progeny, while 35% of the eggs produced by D76N β_2_-m-expressing worms did not hatch (Fig. 5d).

We therefore confirmed that the phenotypic impairment, shown by the movement index parameter, is attributable to a sum of various contributions: defect in the motility, reduction in the lifespan and fertility and larval development impairment of the D76N β_2_-m expressing strain. No significant difference was detected in D76N β_2_-m expressing adult worms in terms of dimensions in comparison to controls (S4).

Experiments performed on extrachromosomal strains expressing WT and D76N β_2_-m confirmed the higher toxicity of the variant compared to the WT form (S5).

### Phenotypic rescue by doxycycline

**Figure 6:**
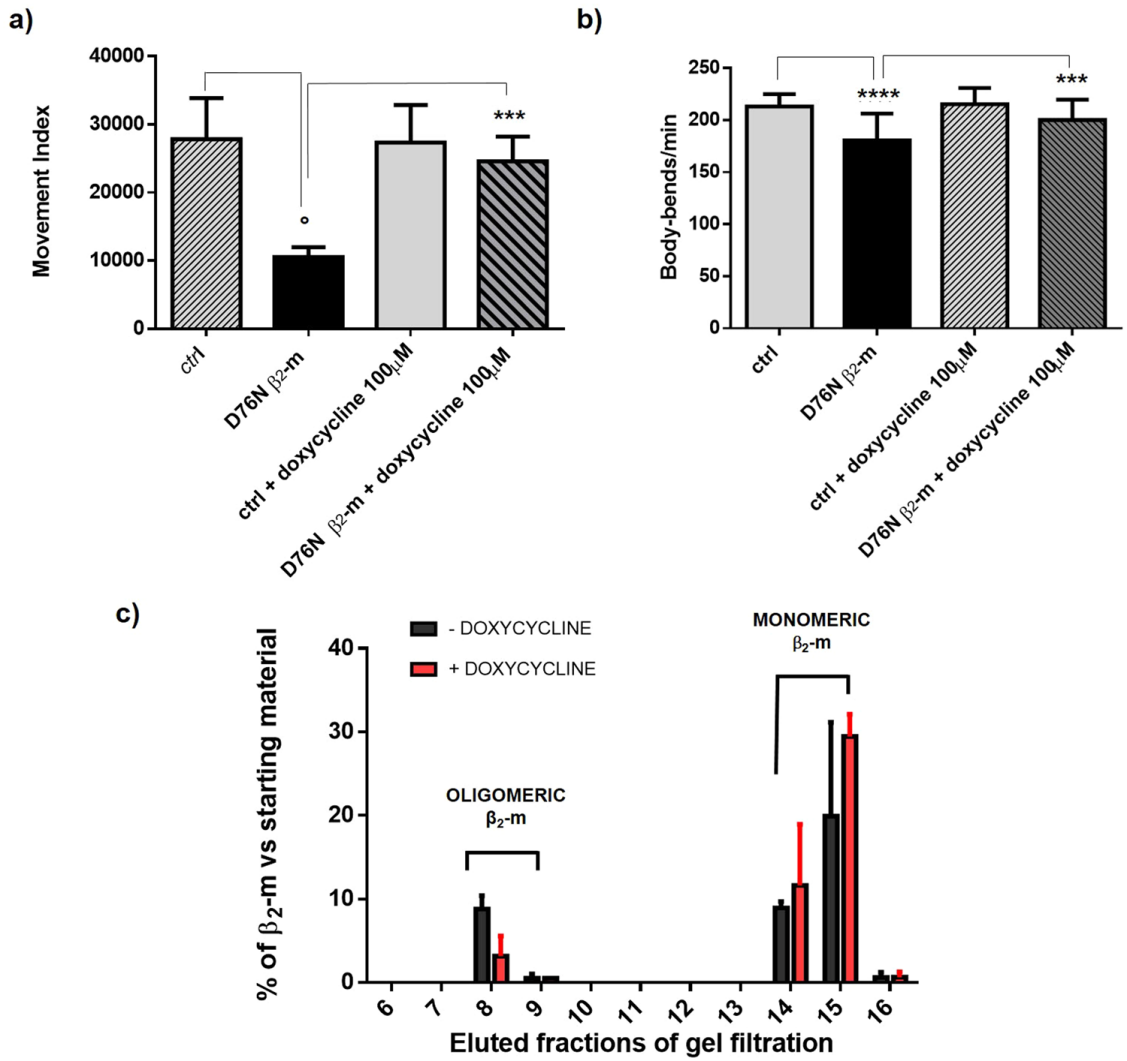
(**a)** Movement index analysis in presence and absence of doxycycline. Three L4 worms/well were placed onto NGM 6-well plates, fed with OP50 *E. coli*, and incubated at 25 °C for six days in presence of 0 or 100 μM of doxycycline from the L4 larval stage. Then the plates with adult worms and progenies were analyzed by INVAPP Paragon software. Three independent experiments were carried out and the results were plotted using GraphPad Prism (v6), ***p < 0.001 vs the untreated control (D76N β_2_-m expressing nematodes) and °p < 0.5 vs. the ctrl worms according to one-way Anova. **(b)** Egg-synchronized control worms (*smg-1*) and D76N β_2_-m expressing worms were placed at 23 °C into fresh NMG plates seeded with OP50 *E*. *coli*. At day 5 of adulthood body bends were scored in liquid. At least three independent assays were performed. Data are mean of number of body bends/min ± SEM; ***p < 0.001 vs the untreated control (D76N β_2_-m expressing nematodes) and ****p < 0.0001 vs the ctrl worms, according to one-way ANOVA (N = 40 animals for each group). **(c)** Percentage of β_2_-m in eluted fractions from gel filtration of D76N β_2_-m expressing worms treated with 0 or 100 μM of doxycycline, is given as ratio of D76N β_2_-m quantity of eluted fraction/soluble fraction starting material quantified from WB bands density related to the monomeric molecular weight (mean ± SEM, n = 2). Uncropped scans of immunoblots are shown in Supplementary Fig. S6. Density of the bands was determined by Image Studio Lite (LI-COR Biosciences).

This novel transgenic *C. elegans* model was used to investigate the phenotypic rescuing capacity of doxycycline, a drug able to inhibit *in vitro* the fibrillogenesis of D76N β_2_-m^17,18^. Nematodes treated with 100 μM doxycycline were analysed after 6 days at 25 °C using the INVAPP/Paragon system showing that the treatment increased the movement index by two-fold, compared to untreated controls (Fig. 6a, ***p < 0.001 vs the untreated control according to one-way Anova). Thus confirming the efficacy of this drug in reducing the cytotoxicity of amyloidogenic proteins also *in vivo*^12,17,19^. In order to analyze only the contribution of the drug on movements, we performed a body bends assay on adult worms that were grown in presence of 100 μM doxycycline. At day 5 of adulthood, we observed a recovery in the motility of D76N β_2_-m worms treated with this drug compared to controls not treated, indicating that doxycycline also has an effect on movement (Fig. 6b, ***p < 0.001 vs the untreated control, according to one-way ANOVA).

To understand the still elusive protective mechanism of doxycycline, we tested in our model the formation of oligomers in the presence of the drug. By using size exclusion chromatography followed by western blot analysis, we successfully observed a reduction of the oligomeric β_2_-m species in nematodes treated with doxycycline (Figs. 6c, S6). As shown in Fig. 6c, the fraction of oligomers is considerably reduced in the D76N β_2_-m worms treated with the drug (Fig. 6c, red bars).

## Conclusions

The *smg*-system enables the inducible expression of D76N β_2_-m, thereby circumventing its lethal toxicity in the very early stages of *C. elegans* development. The novel *C. elegans*’ strain (CPV27) expressing this rare and pathogenic variant of β_2_-m represents the first robust animal model for studying the pathogenic conformer of the β_2_-m protein. Although amyloid deposits were not detected, we have shown that the β_2_-m variant self-aggregates *in vivo* generating soluble, high molecular weight, oligomeric species that are responsible for an abnormal phenotype in our novel *C. elegans* strain.

Our attempts to visualize and localize β_2_-m using anti-β_2_-m antibody tagged with a fluorescent dye were not completely successful (data not shown) but, in order to consider more deeply the pathogenesis of D76N β_2_-m-induced pathological phenotype, it will be important to pursue immunofluorescence studies with improved antibodies.

We demonstrated the capacity of doxycycline to rescue the adverse phenotype resulting from D76N β_2_-m, most likely by interfering with the formation of oligomeric conformers *in vivo*^12,17^.

The INVAPP/Paragon platform is a useful tool for studying the proteotoxicity of this β_2_-m variant. In future, this platform, which tends itself to high-throughput chemical screening, may expedite the search for new drug candidates for the treatment of D76N β_2_-microglobulin related amyloidosis, an unmet clinical need.

## Methods

### Construction of *C. elegans* strain and maintenance

Transgenic animals with thermo-inducible expression of β_2_-m based on the well-characterized *smg* mRNA-surveillance system^13^ were prepared modifying the pPD118.60 plasmid (L3808, a  gift from Andrew Fire, Addgene plasmid #1598; http://n2t.net/addgene:1598; RRID:Addgene_1598), which was then injected in PD8120 *smg-1(cc546) C. elegans* strain, provided by the *Caenorhabditis* Genetics Center (CGC, University of Minnesota, USA). To modify plasmid pPD118.60, oligonucleotides containing compatible sticky ends and encoding the signal peptide of *C. elegans sel-1* (Forward oligo: 5′GCCGCATGATTAAA ACCTATCTGACACTGTTGCTACTAGCAACTCGGCCACGTGTG 3′; Reverse oligo: 5′CTAGCACACGTGGCCGAGGTTGCTAGTAGCAACAGTGTCAGATAGGTT TTAATCATGC3′) (Primm, Milan, Italy) were ligated between the unique *Not*I and *Nhe*I sites in the pPD118.60 plasmid. Amplified cDNA for human D76N β_2_-m was inserted in the engineered plasmid containing *sel-1* signal peptide between the unique *Not*I and *Nhe*I restriction sites. DNA sequencing was carried out to confirm that the sub-cloned plasmid was correct. The plasmid obtained (PAV1) was therefore injected into *smg-1(cc546)* strain as part of a DNA mix containing 30 ng/μl of β_2_-m construct PAV1 together with 40 ng/μl of plasmid p*odr-1::rfp* as co-injection marker (kind gift  of C. Bargmann, Rockfeller University, New York, USA), and extrachromosomal transmitting lines were obtained. A strain expressing WT β_2_-m was also similarly established, inserting amplified cDNA for human WT β_2_-m in the engineered plasmid aforementioned (see S4 for the phenotypic characterization of the strain). Completely stable chromosomally integrated lines expressing D76N variant were subsequently derived after UV irradiation^20^, and one clone named CPV27, was chosen for subsequent analysis. After irradiation, CPV27 strain was back-crossed with *smg-1* ancestral worms in order to remove background mutations arising from the irradiation process. Transgenic worms were thus engineered to express human D76N β_2_-m under the temperature inducible control of the body-wall muscle-specific *myo-3* promoter.

The *smg-1* ancestral strain and D76N β_2_-m strain were grown in Petri dishes on nematode growth medium (NGM) and fed with the OP50 strain of *Escherichia coli*^20^. Age synchronized worms were obtained by bleaching adult nematodes with alkaline solution (500 mM NaOH, 1.5% NaClO) and eggs were isolated and maintained at 16 °C. When they reached the L1 larval stage, the expression of D76N β_2_-m was induced by increasing temperature to 23 or 25 °C.

### Genotype characterization

*C. elegans* DNA was extracted using lysis buffer for DNA extraction (10 mM Tris-HCl pH 8.3, 50 mM KCl, 2.5 mM MgCl_2_, 0.45% Tween20, 0.45% Triton X-100, 200 μg/ml proteinase K) and by incubation for 1 h at 60 °C. To check the presence of the injected plasmid, single worm PCR was carried out using the following primers: L3808ForEst: 5′TGCTATG AAAACGGCACAAA 3′, L3808RevEst: 5′ TTCTTCTTCACGTTCCTCACTG 3′. Expected molecular weight of PCR products was verified by DNA electrophoresis on 1.5% agarose gel. After purification of the PCR products with QIAquick PCR Purification Kit (Qiagen), DNA sequencing was performed (Eurofins Genomics Italy, Milano).

### β_2_-m expression

Worms were collected at the first or fifth day of adulthood, in M9 buffer (45 mM KH_2_PO_4_, 42 mM Na_2_HPO_4_, 85 mM NaCl, 1 mM MgSO_4_ in water) and lysed by sonication in lysis buffer (25 mM Tris-HCl pH 7.5, 5 mM NaCl, 5 mM EDTA, 1 mM DTT, protease inhibitor cocktail Roche Applied Science). For each lysate, equal amounts of total proteins, quantified with the Pierce BCA Protein Assay Kit (ThermoScientific), were loaded onto either a 4–20% Mini-PROTEAN TGX (Biorad) or 8–18% Excel SDS gel (GE Healthcare) for electrophoresis performed under reducing conditions. Proteins were transferred to Immobilon P membranes (Millipore) and blocked with 5% non-fat milk, in tris-buffered saline and Tween 20 (TBS-T), for one hour. Western blots were developed with 4.6 μg/ml rabbit polyclonal anti-human β_2_-m antibody (A0072, Dako) O.N. at 4 °C and 1.3 ng/ml anti-rabbit IgG peroxidase conjugate (A0545 Sigma) for 1 h RT, as primary and secondary antibody respectively. To normalize the content of total protein, western blot was developed with 0.185 μg/ml anti-glyceraldehyde 3-phosphate dehydrogenase antibody (anti-GAPDH selected as loading control, ab181602 Abcam) O.N. at 4 °C, and 1.3 ng/ml secondary anti-rabbit IgG peroxidase conjugate (A0545 Sigma) antibody for 1 h RT. Immunoreactive bands were detected by ECL chemio-luminescence (Millipore), and quantified with Image Studio Lite (LI-COR Biosciences).

### Self-assembly of D76N β_2_-m in CPV27 *C. elegans* strain

A pellet containing transgenic *C. elegans* was resuspended in M9 and lysis buffer for further sonication. After centrifugation at 21,000 *g* for 10 min at 4 °C, the soluble fraction was collected and diluted to 1 mg/ml with water. A single 500 μl sample containing 0.5 mg total protein was loaded into a Superdex 75 10/300 GL gel filtration column equilibrated and eluted with PBS pH 7.5 at a flow rate of 0.5 ml/min using an Akta Pure FPLC. Fractions of 1 ml were collected and analysed by 8–18% SDS-PAGE and immunoblotted as above.

### Larval growth and motility automated assay (INVAPP/Paragon system)

Three synchronized nematodes, at their L4 larval stage, were picked onto NGM 6-well plate and incubated at 25 °C. After six days at 25 °C, plates were imaged using a fast high-resolution camera (Andor Neo, resolution 2560 × 2160, maximum frame rate 100 frames/s) with a line-scan lens (Pentax YF3528) as previously described^16^. Plates were placed in a holder built into the cabinet and imaged from below. Movies were captured using μManager and analyzed with a set of MATLAB scripts (https://github.com/fpartridge/invappparagon^16^). Briefly, this involved calculating the variance through time for each pixel. Pixels whose variance was above the threshold (typically those greater than one standard deviation away from the mean variance) were considered ‘motile’. The ‘motile’ pixels were counted and a movement score generated for each well/plate.

### Gene silencing by RNA interference (RNAi)

The RNAi experiments were performed using a feeding procedure^21^. *C. elegans* were fed with HT115 (DE3) bacteria expressing dsRNA targeting β_2_-m. Human β_2_-m cDNA was inserted in the L4440 vector (kind  gift of A.Fire, Stanford University, USA) between *Kpn*I and *Sac*I restriction sites to obtain plasmid PAV2. HT115 *E. coli* bacteria were transformed with PAV2 plasmid and then cultured onto LB plates with 100 μg/ml ampicillin and RNAi production was induced by the addition of 1 mM IPTG. Control HT115 bacteria were prepared after transformation with the empty L4440 vector. Synchronized nematodes at the L4 larval stage were cloned onto NMG plates seeded with RNAi bacteria or the control. Plates were imaged after 5 days at 25 °C using the INVAPP/Paragon system and motility scored. Animals were also collected for western blot analysis.

### Body bends assay

Body bends assays were performed using a stereomicroscope (M165 FC Leica) equipped with a digital camera (Leica DFC425C and SW Kit). Worms at day 1, 5 and 6 of adulthood incubated from L1 larval stage at 23 °C in presence or absence of 100 µM of doxycycline were picked and transferred into a 96-well microtiter plate containing 100 µl of M9 buffer. The number of left-right movements in a minute was recorded. In order to remove the progeny, worms were transferred onto fresh NGM plates every day.

### Life-span assay

Forty synchronized adult worms maintained at 16 °C were upshifted to 23 °C at the larval stage L1. Every day, they were transferred onto a freshly prepared NGM plate until the cessation of egg-laying to avoid the overlapping of generations. Viability was monitored until all worms were reported dead when they failed to display touch-evoked movement.

### Brood size assay and embryonic lethality

Synchronized populations, were grown at 16 °C until their first larval stage, and then upshifted to 23 °C. Afterwards L4 transgenic hermaphrodites were individually cloned onto agar plates maintained at 23 °C and subsequently transferred onto fresh plates at 8-to-16 hour intervals until the second day of adulthood. Then total number of eggs laid in 48 hours was scored and embryos were considered dead if they had not hatched after 24 h at 23 °C. The brood size of each animal is the sum of non-hatched and hatched progeny. Embryonic lethality is the number of non-hatched embryos divided by the sum of non-hatched and hatched progeny.

### Doxycycline treatment

Synchronized nematodes at L4 larval stage, incubated at 25 °C from the L1 larval stage to allow protein expression, were placed into NGM agar plates seeded with HT115 E. *coli* resistant to tetracycline and in the presence of 0 or 100 μM of doxycycline. Plates were imaged six days later using the INVAPP/Paragon system and motility scored. Worms were also collected and lysed in order to perform size-exclusion chromatography and western blot analysis as reported before.

### Statistical analysis

Data were analysed using independent Student’s t-test or one-way ANOVA test with GraphPad Prism 6.0 software (CA, USA). A p value under 0.05 was considered statistically significant.

## Supplementary information

Supplementary material
