# Supplementary material for: C. elegans expressing D76N β2-microglobulin: a model for in vivo screening of drug candidates targeting amyloidosis
Source: Sci Rep. 2019 Dec 27;9:19960. doi: 10.1038/s41598-019-56498-5 (PMC6934621; doi:10.1038/s41598-019-56498-5)
Supplement: Supplementary file 1 — Supplementary material [file 41598_2019_56498_MOESM1_ESM.pdf]

C. elegans expressing D76N β_2-microglobulin: a model for _in vivo_ screening of drug candidates targeting amyloidosis.

Giulia Faravelli[1*+], Sara Raimondi[1+], Loredana Marchese[1], Frederick A. Partridge[2], Cristina Soria[1], P. Patrizia Mangione[1,3], Diana Canetti[3], Michele Perni[4], Francesco A. Aprile[4], Irene Zorzoli[1], Elia Di Schiavi[5], David A. Lomas[2], Vittorio Bellotti[1,3], David B. Sattelle[2] & Sofia Giorgetti[1*].

[1]Department of Molecular Medicine, Institute of Biochemistry, University of Pavia, 27100 Pavia, Italy.
[2]Centre for Respiratory Biology, UCL Respiratory, Division of Medicine, University College London, Gower Street, London, WC1E 6JF, United Kingdom.
[3]Wolfson Drug Discovery Unit, Centre for Amyloidosis and Acute Phase Proteins, University College London, London, UK.
[4]Centre for Misfolding Diseases, Department of Chemistry, University of Cambridge, Cambridge, CB2 1EW, UK.
[5]Institute of Biosciences and Bioresources (IBBR), CNR, 80131, Naples, Italy.
*giulia.faravelli01@universitadipavia.it, s.giorgetti@unipv.it
[+]these authors contributed equally to this work

Faravelli and Raimondi *et al*. Supplementary Figure S1

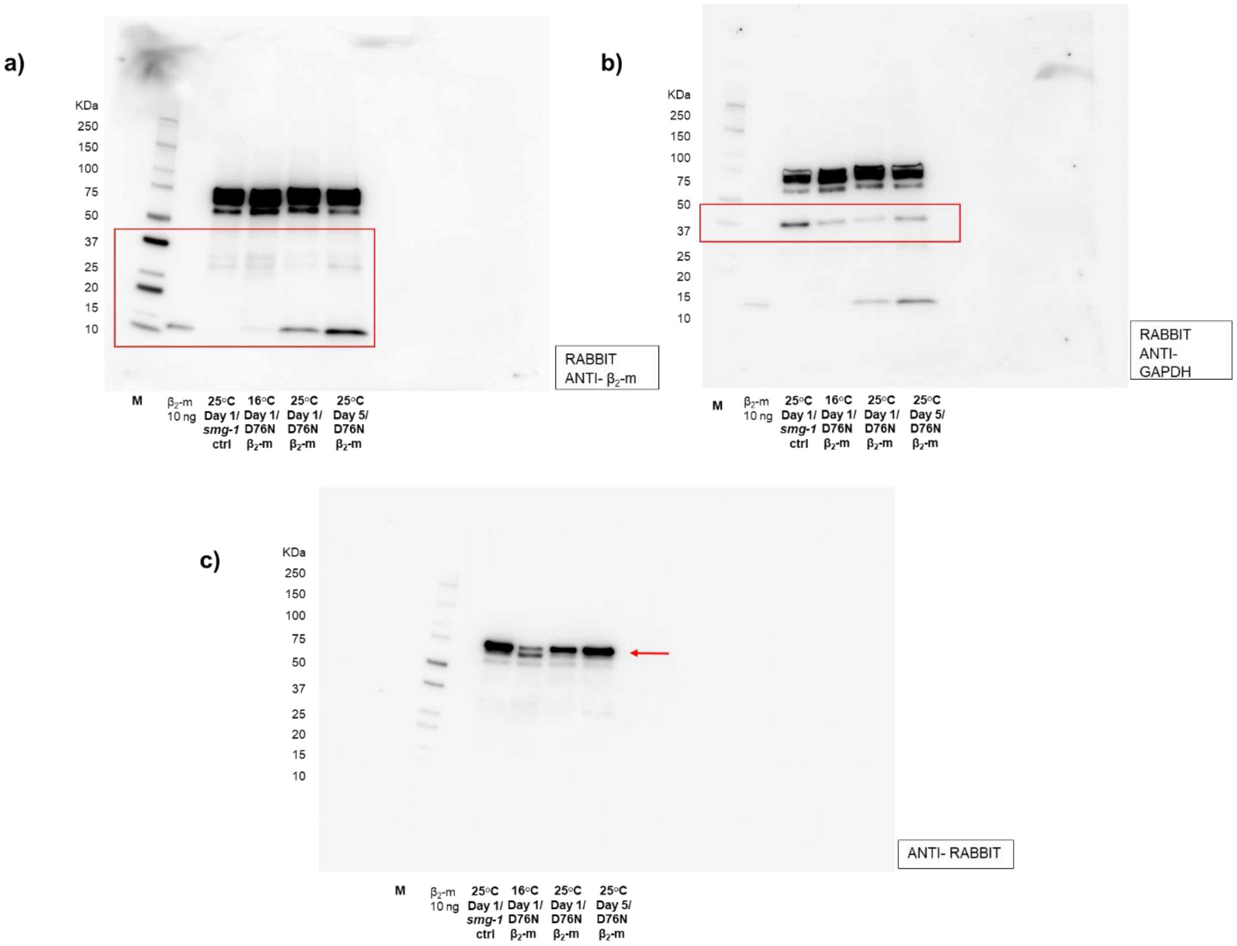

Supplementary Figure S1. **Western blots for detection of a) β_2-m and b) GAPDH.** We probed the membrane with rabbit polyclonal anti-human β_2-m antibody (A0072, Dako) **a)** and with both rabbit polyclonal anti-human β_2-m antibody and anti-glyceraldehyde 3-phosphate dehydrogenase antibody (anti-GAPDH selected as loading control, ab181602 Abcam) as primary antibodies **b)** and anti-rabbit IgG peroxidase conjugate (A0545 Sigma) as secondary antibody. Portions (red squares) are used in Figure 1c. (M= Molecular weight standard: Precision Plus Western C, BioRad). **c)** Western blot analysis was carried out on the same samples and detected by using only the polyclonal swine anti-rabbit secondary antibody. This analysis shows that the aspecific band (red arrow) seen at 75 kD is attributable to the cross-linking of the secondary antibody.

Faravelli and Raimondi *et al*. Supplementary Figure S2

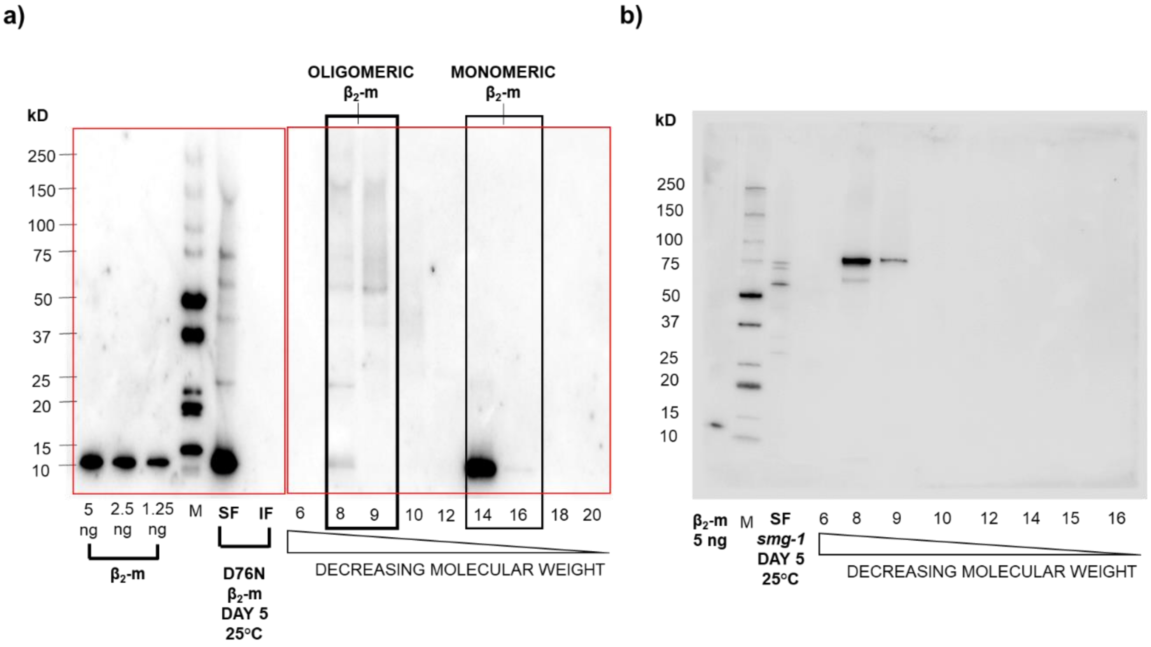

Supplementary Figure S2. **a) Western blot for detection of β2-m.** Portions (red squares) are used in Figures 2a and 2c. (M= Molecular weight standard: Precision Plus Western C, BioRad). **b)** The soluble fraction of *smg-1* control worms was analysed by gel filtration and by western blot, as reported in the main text, as negative control.

Faravelli and Raimondi *et al*. Supplementary Figure S3

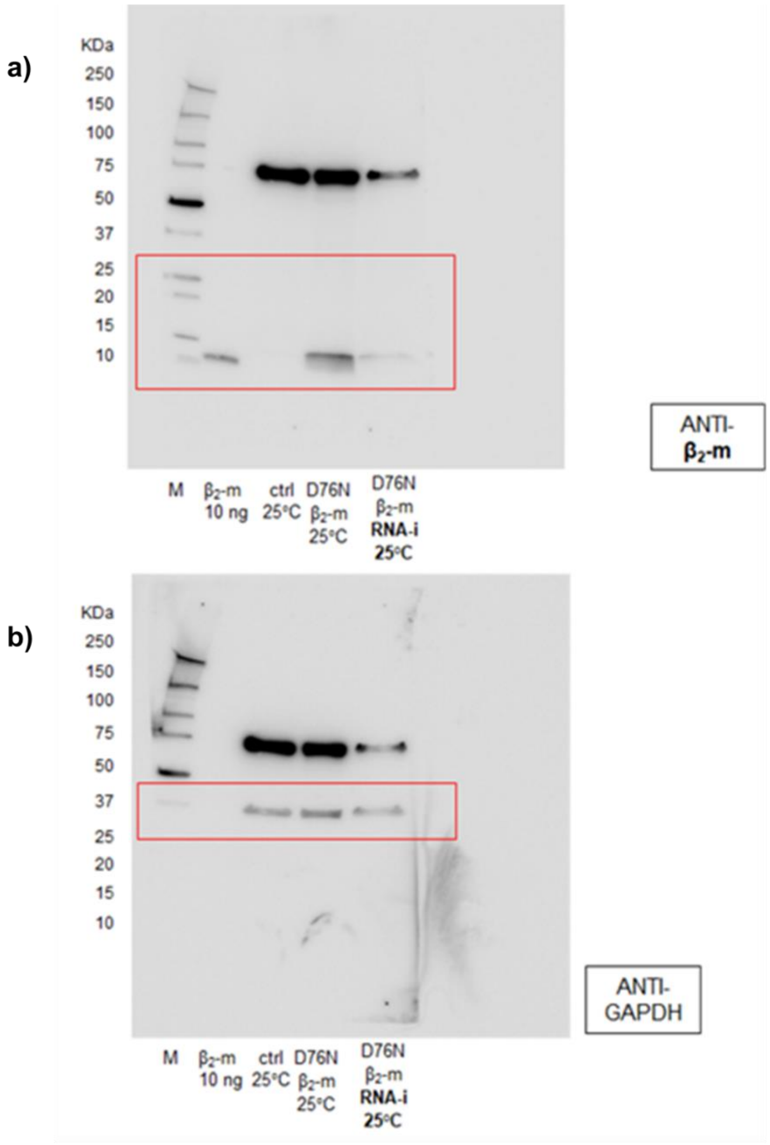

Supplementary Figure S3. **Western blots for detection of a) β2-m and b) GAPDH.** Portions (red squares) are used in Figures 4a and 4b. (M= Molecular weight standard: Precision Plus Western C, BioRad).

Faravelli and Raimondi *et al*. Supplementary Figure S4

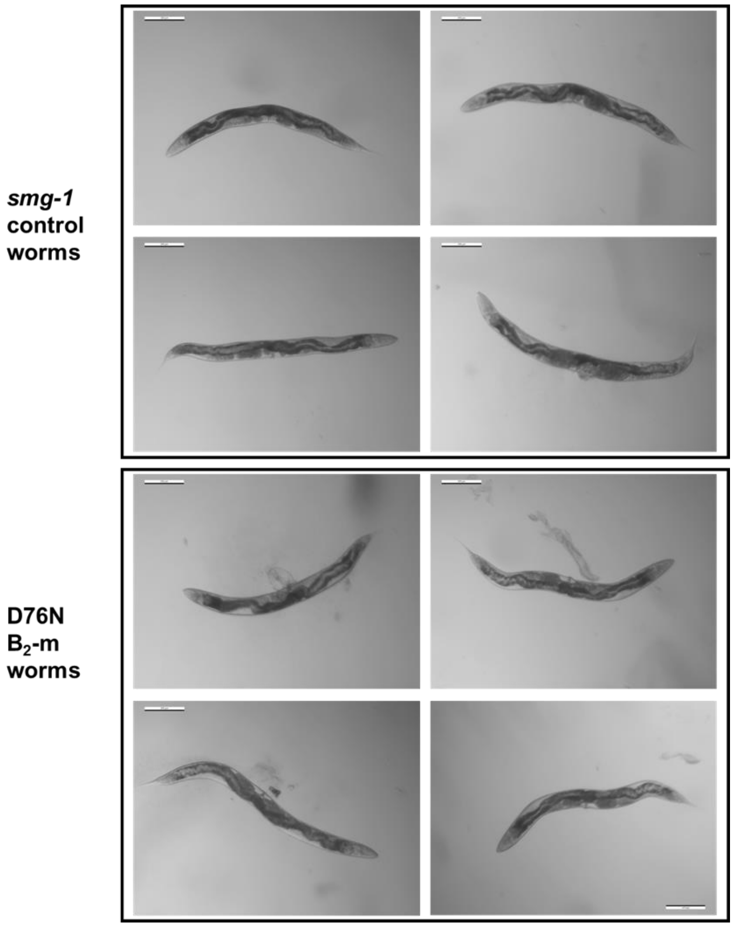

Supplementary Figure S4. Bright-field images of D76N β_2-m. 4 CPV27 worms (lower panel) and *smg-1* controls (upper panel) at day 4 of adulthood were incubated from the L1 larval stage at 23°C. Images were obtained with an inverted contrasting microscope (DM IL Leica Microsystems) and a CDD camera. Scale bars: 200 μM.

Faravelli and Raimondi *et al.* Supplementary Figure S5

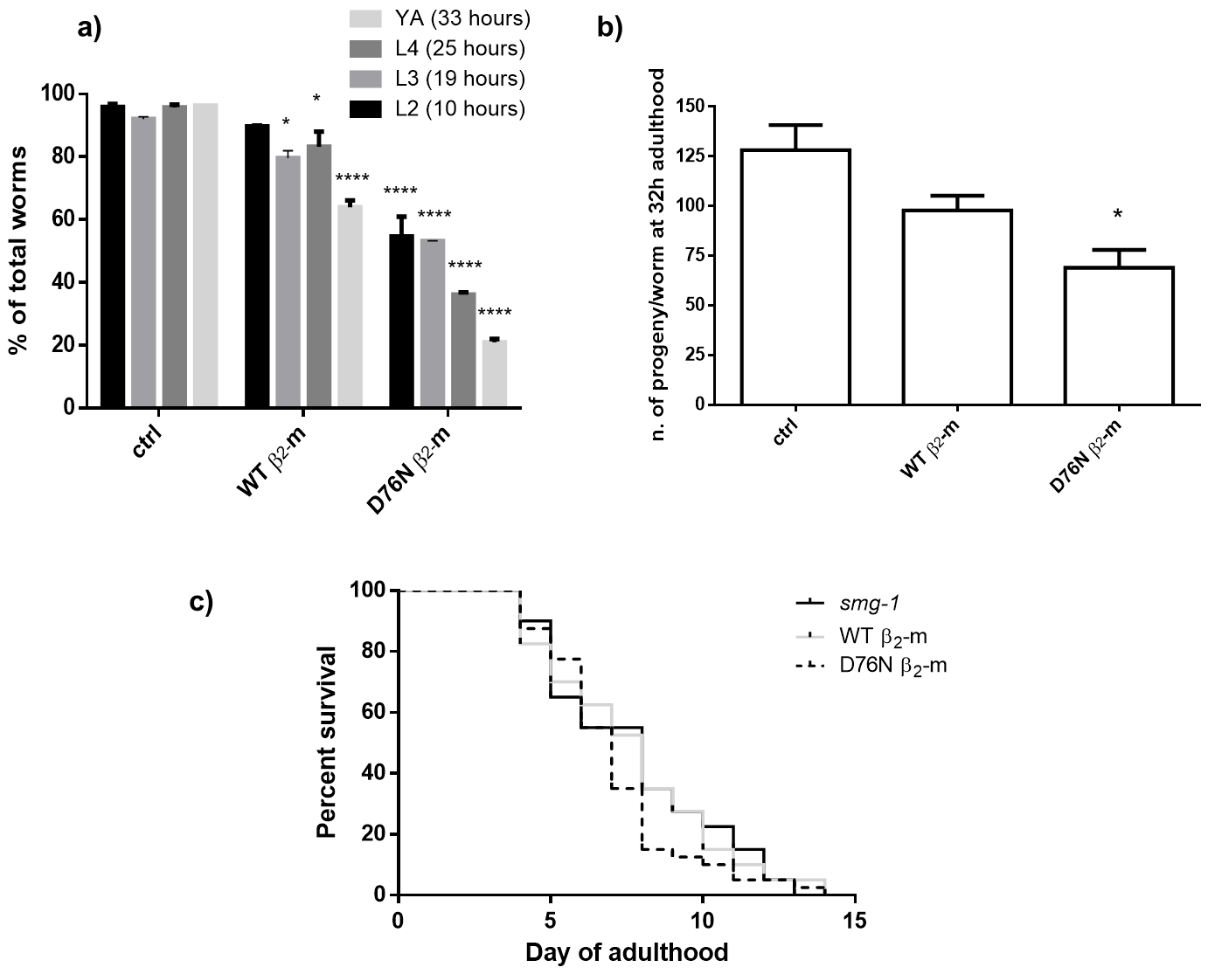

Supplementary Figure S5. Characterization of the phenotype of WT and D76N β_2-m expressing strain. a) Larval growth of control non-transgenic animals, WT and D76N β_2-m expressing strains exposed to 25°C from the first larval stage. All animals are mutated in *smg-1 (cc456)*. One hundred synchronized L1 nematodes were placed into fresh NMG plates seeded with OP50 as food and the number of L2, L3, L4 and young adult worms was scored after 10, 19, 25 and 33 hours, respectively. Data are expressed as percentage of total worms in the plate at each time point and are given as mean of two independent experiments (N = 200). Error bars represent the SD, *$p<0.05$ and ****$p<0.0001$ vs. the control strain (*smg-1*) according to one-way ANOVA. **b)** Total eggs deposition for non-transgenic animals, WT β_2-m and D76N β_2-m strains maintained at 23°C for 32 hours after the upshift of the temperature from L3 larval stage. N = 20 nematodes in each graph. Error bars represent the SD, *$p<0.05$ vs. the control strain (*smg-1*) according to one-way ANOVA. **c)** Kaplan–Meier survival curves of control nematodes, WT and D76N β_2-m expressing strain at 25°C. Data are expressed as mean of two independent experiments (N=40 animals for each group). The median survival is about 8 days for control strain and WT β_2-m expressing worms, while is 7 days for worms expressing the D76N variant.

Faravelli and Raimondi *et al*. Supplementary Figure S6

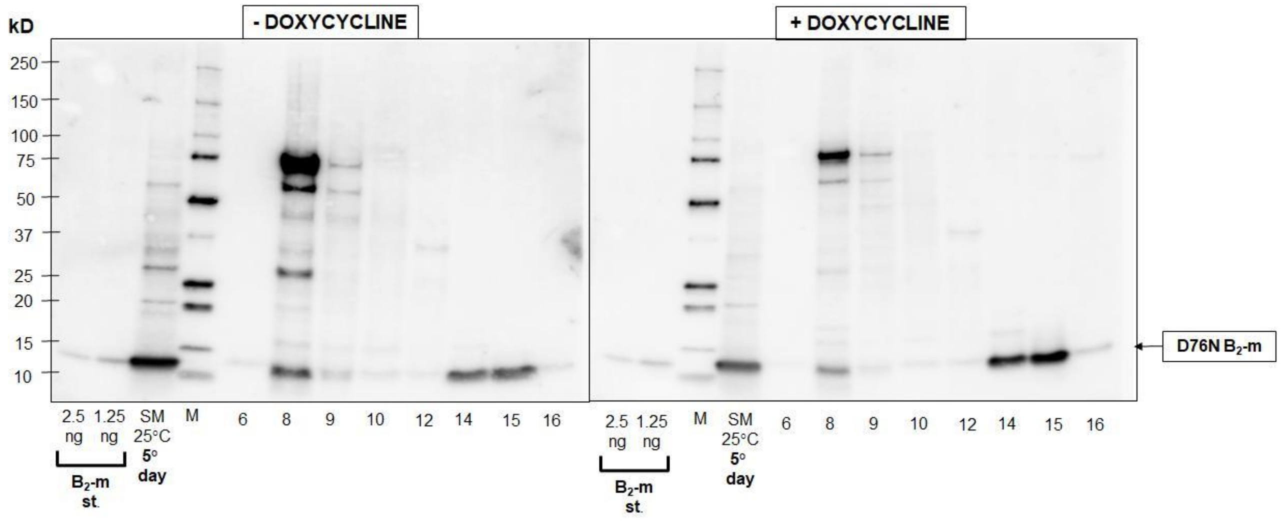

Supplementary Figure S6. Effect of doxycycline on D76N β2-m expression in CPV27 strain. Western blot analysis of gel filtration eluted fractions of CPV27 worms' lysates treated in the absence or in the presence 100 µM of doxycycline. Size-excluded fractions (6-16) were resolved via 8-18% SDS PAGE, blotted and detected with a polyclonal anti-β2-m antibody (DAKO) as described in the Methods section in the main text. (M= Molecular weight standard: Precision Plus Western C, BioRad).
